# Developing and Implementing a Community-Based Model of Care for Fibromyalgia: A Feasibility Study

**DOI:** 10.1155/2017/4521389

**Published:** 2017-07-16

**Authors:** Michelle Teo, Bindu Mohan, Nelly D. Oelke

**Affiliations:** ^1^Balfour Medical Clinic, 1496 Balfour St, Penticton, BC, Canada V2A 4Z1; ^2^Penticton Regional Hospital, 550 Carmi Avenue, Penticton, BC, Canada V2A 3G6; ^3^Department of Medicine, Faculty of Medicine, 2775 Laurel Street, 10th Floor, Vancouver, BC, Canada V5Z 1M9; ^4^School of Nursing, University of British Columbia Okanagan, BC Support Unit Interior Regional Centre, FHSD, Kelowna, BC, Canada V1V 1V7; ^5^Department of Community Health Sciences, Cummings School of Medicine, University of Calgary, Calgary, AB, Canada

## Abstract

**Background:**

Fibromyalgia (FM) is a complex disease posing challenges for primary care providers and specialists in its management.

**Aim:**

To evaluate the development and implementation of a comprehensive, integrated, community-based model of care for FM.

**Methods:**

A mixed methods feasibility study was completed in a small urban centre in southern British Columbia, Canada. Eleven adults with FM and a team of seven health care providers (HCPs) participated in a 10-week intervention involving education, exercise, and sleep management. Monthly “team-huddle” sessions with HCPs facilitated the integration of care. Data included health questionnaires, patient interviews, provider focus group/interviews, and provider surveys.

**Results:**

Both patients and HCPs valued the interprofessional team approach to care. Other key aspects included the benefits of the group, exercise, and the positive focus of the program. Effectiveness of the model showed promising results: quality of care for chronic illness, quality of life, and sleep showed significant (*P* < 0.05) differences from baseline to follow-up.

**Conclusions:**

Our community-based model of care for FM was successfully implemented. Further testing of the model will be required with a larger sample to determine its effectiveness, although promising results were apparent in our feasibility study.

## 1. Introduction

Fibromyalgia (FM) is a chronic musculoskeletal pain disorder affecting 5% of patients presenting to primary care. It is commonly associated with other symptoms such as fatigue, sleep disturbances, mood and cognitive changes, headaches, and irritable bowel syndrome, alongside comorbidities such as rheumatoid and inflammatory arthritis [1]. Its prevalence is higher among women (7.7%) than men (4.9%) [2]. FM has significant impact on the physical and mental functioning and quality of life of those affected [3, 4]. Dysfunctional central pain processing is thought to be one of the key underlying mechanisms along with other factors such as mental and physical trauma and genetic predisposition [1]. With complex presentations and lack of diagnostic investigations, the diagnosis and treatment of FM are both challenging, varied, and time-consuming. The lack of a defined pathway to manage this chronic condition results in lengthy wait times to see specialists, poly pharmacy of analgesic medications, lack of community resources to support self-management, rising disability, and thereby increased socioeconomic burden. Barriers to the management of FM include lack of successful treatments, lack of collaboration of HCPs, and absence of emotional support for FM patients. Patient surveys show little satisfaction with pharmacological intervention alone [5, 6]. However, in recent years, treatment guidelines for FM have looked towards a more holistic approach. An interprofessional approach combining pharmacological interventions with aerobic exercise, patient education, and cognitive behavioural therapy has shown great promise in randomized trials [7–14]. Evidence-based guidelines from Canada, Germany, Israel, and the European League against Rheumatism compare similarly for nonpharmacological treatment as the first line of treatment for FM management [15]. The 2012 Canadian guidelines on the diagnosis and treatment of FM recommend multimodal management and active patient involvement with a view to enhancing function, encouraging healthy lifestyle practices, and continuing employment [1].

Research on the establishment and sustainability of interprofessional care in smaller communities is minimal [16]. Our feasibility study was conducted in the small urban centre of Penticton, located in the southern interior of British Columbia, Canada. A comprehensive interprofessional community-based model of care was developed in an effort to address the needs of FM patients. The primary goal of this study was to understand the structures and processes of an integrated community-based model of care and to test its development and implementation in this community. The secondary aim was to determine if such a model of care would be beneficial in improving the quality of life of FM patients and to assess the potential sustainability of this particular model in a community setting.

## 2. Materials and Methods

This prospective, mixed methods [17] feasibility study used a pre-post design [18] supplemented with interviews with patients and focus groups/individual interviews with HCPs. The study was designed with the aim of providing a platform for patients that combined education and practical training in the long term and effective management of FM. The research protocol received approval from the University of British Columbia Okanagan (UBCO) and Interior Health Research Ethics Boards.

### 2.1. Sample and Setting

Penticton is a small urban centre located in southern interior British Columbia with a population of just over 33,000 people, serving an additional population of approximately 80,000 people [19]. In 2011, 26% of Penticton's population was 65 and over [20]. Penticton has a regional hospital with 137 beds. Primary care and specialty medical services are offered in the city along with other community-based services.

Eleven patients with FM from Penticton and the surrounding areas, who met eligibility criteria, were enrolled in the study. Eligibility criteria included the following: being 19 years of age or over; having a diagnosis of FM; living in Penticton or surrounding areas; and committing to participating in all aspects of the intervention. Those patients who were pregnant or had a severe and/or chronic medical condition (e.g., schizophrenia and heart disease) that would impact their participation in the FM program were not included in the study. Seven HCPs completed the HCP survey and six HCPs participated in a focus group or individual interview.

### 2.2. Outcomes

The primary outcome measure was patient and HCP perspectives on the model of care. Secondary outcomes included the improvement in patient health over the intervention period as measured by the nine healthcare questionnaires.

### 2.3. Intervention

Based on the existing models of multidisciplinary treatment for FM, a team of HCPs was put together for this pilot intervention [16]. A family physician was involved in the planning stages of this pilot study. Studies on multidisciplinary interventions for FM typically run between 2 and 12 weeks, with variable success [12, 16]. The duration of this intervention was chosen to be 10 weeks as it was considered by our HCPs as the optimal time required for a measurable change to occur. The 10-week intervention combined education, exercise training, sleep management, and pharmacological interventions provided by a team of seven HCPs: a rheumatologist, rheumatology nurse, physiotherapist, exercise therapist, physiotherapist specializing in chronic pain management, psychiatrist, and dietitian. Details of the intervention are provided in Table 1. HCPs also participated in monthly “team huddle” sessions via teleconference to discuss issues with the intervention as well as review patient progress. The estimated cost per patient for the 10-week intervention was approximately $200 CAD. This covered the physiotherapist, exercise therapist, pain professional, and dietitian. Consultation fees for the rheumatologist, rheumatology nurse, and psychiatrist were covered through our provincial Medical Services Plan.

The exercise and education sessions were held at the Penticton Community Centre gym space and the psychiatric assessments and follow-up sessions were held at the private clinic of the psychiatrist involved in the study. There was no cost to patients for the services provided.

### 2.4. Data Collection

Data were collected at baseline (patient demographics, nine health care questionnaires [see Table 2]) and at three months (interviews with patients, nine health questionnaires, focus group/interviews, and survey with HCPs), at six and twelve months (nine health questionnaires) after initiation of the 10-week intervention.

Qualitative data collection included semistructured in-depth interviews of patient participants, on a one-to-one basis. Interviews were conducted either in person (*n* = 8) or over the phone (*n* = 3). The interviews consisted of open-ended questions regarding patient experience and their perspective of the model of care. The monthly “team-huddle” sessions were attended by most of the HCPs and provided an opportunity for the HCPs to address concerns and discuss changes to the model of care. The “huddles” also provided an opportunity for HCPs to discuss specific patient/clinical concerns. A focus group was conducted following the final “team huddle,” to gather feedback from the HCPs on the model of care and the changes they recommended (*n* = 3). Due to conflicting work schedules of various HCPs, some of the HCPs were interviewed by phone (*n* = 3). A total of 6 HCPs provided feedback on the model of care. All interviews and the focus group were digitally recorded and transcribed verbatim by an experienced medical transcriptionist. Field notes collected during the interviews and focus group session were also transcribed and used in data analysis.

### 2.5. Data Analysis

Quantitative data from the health questionnaires were divided into appropriate dimensions for each of the questionnaires to facilitate analysis. Central tendencies such as mean, median, and standard deviations were analyzed for each dimension. The nonparametric test, Wilcoxon's signed ranked test, was used to compare baseline and postintervention changes for each parameter. 95% confidence intervals between pretreatment and posttreatment were considered to be significant. SPSS™ software (version 23) was used to carry out analysis on the quantitative data.

Qualitative data analysis was carried out using NVivo 11™ software. Data was prepared for this purpose by entering transcripts and field notes into the NVivo software program. The analysis involved an iterative process of carefully going through the transcripts of each interview, identifying evolving concepts central to the questions of the project and coding them. Finally interviews and codes were collectively examined to establish relationships and identify broad themes. Triangulation of data from these multiple sources improved the validity of this study [33, 34].

## 3. Results

The age range of our 11 patients was between 39 and 79 years with a mean of 55.36 (±11.87). Patients had FM for an average of 10.71 (±7.78) years. Employment and comorbidities are shown in Table 3. The average number of sessions missed per patient over the 10 weeks was 4.36. The minimum number of sessions missed was 1 and the maximum was 8. Five of the 11 participants attended all of the sessions. The patient demographics represent the typical FM patient cohort, characterized by a myriad of heterogeneous comorbidities.

### 3.1. Primary Outcome Measure

Major themes were identified from patient interviews and HCP interviews/focus groups. These themes will be discussed in the following sections.

#### 3.1.1. Model of Care

The program focused on providing a small group of FM patients the education, emotional and social support, and clinical intervention in a structured yet comprehensive manner. Both patients and HCPs identified the interprofessional team, “group” approach, education, exercise, and “team huddles” as valuable components of the model of care. Prior to participating in the intervention most of the participants were unaware of others with FM and were apprehensive of discussing their condition openly. Although they took time to build relationships, they quickly bonded and were able to connect with each other as they shared similar experiences. Being part of a group that shared a common goal facilitated patients' sense of commitment, accountability, purpose, and support:* “what I liked most was…being in a group of people that are dealing with the same issues…I liked the support. I think it was extremely important” *(Participant 05). The model of care provided patients with a novel experience, giving them a chance to learn, share their grievances, communicate freely, and contribute effectively.

The participants also greatly benefited from the educational sessions in pain, fibromyalgia, sleep, and coping strategies. The weekly sessions on FM helped reinforce ideas:* “she did some really good group sessions about myths of fibromyalgia, which I thought was very helpful…they had a session where we could ask them any questions we wanted” *(Participant 09). Overall these educational sessions enabled patients to raise their awareness, change the perceptions of their condition, and facilitate management of their symptoms.

The exercise sessions were regularly supervised by the exercise therapist and physiotherapist; they were tailored to individual needs and were goal-orientated. The participants were able to overcome their fear of exercise-induced exacerbation of FM, learn proper exercise techniques, and adapt it to their lifestyle:

I was always active before but when I got sick I couldn't be…I've been trying to get more active and get an exercise program going. I couldn't do it by myself. I always would end up with a massive flare-up. And I feel stronger now. I feel better…now that I have a plan and I can go forward with this. (*Participant*  02)

The HCPs monthly “team huddles” proved efficient in identifying patient care issues which were addressed quickly, although continuous improvement to sessions would be helpful. Clear direction and expectations for both patients and providers would assist in avoiding confusion or disappointment. Both participants and providers suggested adding a mental health counsellor to the team, particularly earlier in the sessions, to aid the group in connecting and assist with grief counselling, anxiety, and anger management:

They seem to really need someone to talk to…whether it be like a group counselling session in the beginning to kind of have them get bonding sooner…but I think it's still needed no matter what age we are, just to break down some barriers and get people opening up. (*Participant*  *X*05)

Finally, the length of some of the sessions will need to be evaluated as for some participants this was an issue.

#### 3.1.2. Challenges

Through the model, the participants were able to voice some of the challenges they faced as a result of their chronic illness. These included lack of support in the community, inability to cope with chronic grief, overcoming challenges posed by FM, and over prescription of medications. Patients were often left to manage their own symptoms, most over many years, and received little support and intervention from HCPs. For many, participating in the model of care included hope for treatment that may be helpful to manage their FM.

Participants also spoke of their loss of having to deal with a new unexpected life. They grieved for their past life; with a diagnosis of FM, they were unable to engage in many activities and had to deal with chronic pain, and other symptoms such as depression:* “we've lost our old lives. There's a lot of things to grieve” *(Participant 03).

The participants also spoke of the frequent aggravation of symptoms, which is commonly associated with FM. The fatigue, flare-ups, lack of motivation, and emotional draining made it difficult for them to participate in physical activity. This chronic inactivity became a vicious cycle and, without support, it was difficult to break:* “days that we have this program, I don't plan anything else. Because that's about as much as I can do in a day…so, that's the biggest challenge is just getting here. Some days you just don't feel well enough”* (Participant 03).

Most participants suffered from multiple comorbidities and were on several medications for sleep, depression, and other health conditions. HCPs noted that there was a lack of an organized treatment plan for these patients. Making changes to existing mood stabilizing or sleep aid therapies was challenging for the psychiatrist during the study, as many of these medications were initiated by other HCPs. Many patients, however, benefited from these changes. For example, for some patients, reducing their medications improved their sleep, while, for others, it did not make a difference and for some made it worse:* “he cut my medicine in half, my night time medication in half…which enabled me to sleep better. Instead of giving me a sleep aid, he cut mine in half, which was tremendous” *(Participant 09).

#### 3.1.3. Positive Changes

All program components were perceived to encourage participants to develop a positive and healthy attitude assisting them to make important life style changes:* “I think the main thing that has changed is I feel better emotionally…so, I've got a better attitude towards my pain. It's not getting me down so much” *(Participant 07). Another participant commented on her lifestyle changes:* “changing my eating habits. I've lost five pounds over the three months and the doctor's reduced my diabetes medication by half” *(Participant 08). HCPs also noticed the positive environment: “*The patients seemed more optimistic than they were before...and they also seemed to be more knowledgeable about different techniques that they could use to both cope with the pain and to try to recover function” *(Participant X06).

#### 3.1.4. Long-Term Sustainability

HCPs unanimously agreed that this model of care for FM patients had strong potential for long-term sustainability. Our small urban centre was well situated in terms of facilities and the presence of these various HCPs:

These are already available resources that the community offers. The only difference is that they're being done in a collaborative fashion, so I think there is a lot of potential for this to be sustainable and to be able to spread this to other communities, any communities big or small, especially with the involvement of telemedicine now in rural communities. (*Participant*  *X*01)

A strong partnership between the HCPs, the health authority, and the community will be necessary to implement this model of care on a broader scale.

### 3.2. Secondary Outcome Measure

**Figure 1:**
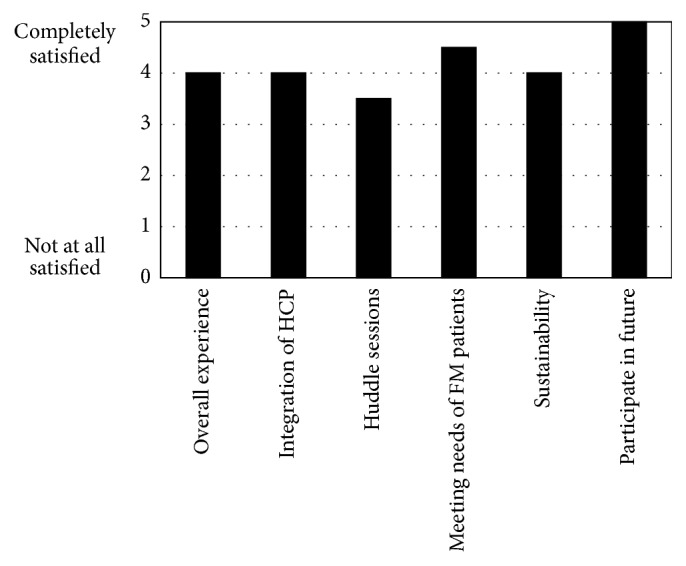
Survey conducted with HCPS on their views of the model of care.

The HCP survey showed overall satisfaction with the model and willingness to participate in future (median score 3.5–5) (Figure 1).

Patient health care questionnaires used as a measure of secondary outcomes of the study demonstrate a trend to improvement in a number of parameters such as quality of care received, quality of life, sleep modalities, and fatigue, as is shown in Table 4. This improvement was seen over the 10-week period of the intervention.

### 3.3. Discussion

The feasibility study discussed above, to establish a comprehensive, community-based model of care for FM patients, was successfully completed in the small urban centre of Penticton. A small cohort of patients diagnosed with FM and an interprofessional team of health care providers participated in this feasibility study. As with other multidisciplinary trials, a range of qualitative tools and quantitative tools were adopted to evaluate the outcomes of the study [35–38].

Results from the data collected demonstrate the success of the model of care and positive impact it had on the FM patient cohort. Qualitative data obtained from patient and HCP interviews highlighted the day-to-day challenges faced by FM patients and the positive impact this model of care had on this cohort. The educational sessions coupled with the exercise sessions helped change patient perception of their condition and adopt more healthy lifestyles. The small group size also impacted the ability of the patients to better connect with each other and share their experiences. Furthermore, the group approach provided much needed support for the participants. The health care providers unanimously agreed on the novelty of the “model of care” and its ability to address the needs of the FM patient population in the community. These findings are corroborated by the literature with other similar models of care [9, 12]. The “team-huddle” sessions conducted between health care professionals aimed to truly integrate patient management as quickly and efficiently as possible. Quantitative data, collected from the patient health care questionnaires, are also suggestive of a trend to improvement over the 10-week intervention period.

This study also highlighted the challenges concurrent with the establishment of this model of care in a community-based setting. The establishment and sustainability of this model are dependent on factors such as the consistent source of funding, community support, a dedicated team of HCPs, and a strong partnership with the local health authority. Group sessions, utilizing available health care resources and community facilities, will assist in keeping costs to a minimum and ensure long-term sustainability. Our model therefore has great potential at being a sustainable form of care with effective support for the FM population in the community.

The results from our study are well aligned with results from other multidisciplinary trials involving exercise and education along with pharmacological interventions [11, 39–42]. As demonstrated by other studies, the patients in this study found the education and exercise sessions of most value in helping them cope with their condition [43, 44]. In comparison to previously conducted studies where interprofessional treatment models were provided in tertiary centres and outpatient clinics [12, 39–41], our study achieved the same at a community level with the aim of establishing long-term sustainable care. The novelty of our model of care is that, apart from providing care by an interprofessional team at a community level, it also focuses on fostering an integrated, collaborative approach between HCPs in the patient care process, which is lacking in other studies. It is evident from the large number of studies being conducted that the concept of interprofessional care is being widely accepted by family physicians, rheumatologists, and other clinicians in the management of FM [45, 46].

The outcome measures used to determine effectiveness of therapies vary between studies. In a recent multidisciplinary study on FM self-management, Bourgault et al. [12] reported that although there was no change in pain intensity levels at the end of the intervention period, there was a significant improvement in patient perception of quality of life and perceived pain levels. The authors suggested moving away from conventional pain intensity measurements and utilizing scales that better portray patient experience and quality of life. Our feasibility study shows promising results in using different measures of patient experience and quality of life.

### 3.4. Strengths and Limitations

Several strengths and limitations of the study are apparent. The model was made possible by the involvement of a team of dedicated HCPs. The study design involved collection of data via multiple methods (healthcare questionnaires, surveys, focus groups, and interviews). The data obtained was subject to both qualitative and quantitative data analyses to provide internal validity to the findings. Both participant and HCP perspectives on the model of care were obtained as a result of the study design.

Despite these strengths, limitations of the study are noted. First, a relatively small sample size could have impacted the quantitative data obtained and made generalizability of the results more limited. Second, patient participants were primarily an aging population with significant comorbidities making the treatment outcomes more challenging. Third, some patients were receiving preexisting treatment for sleep and other psychiatric disorders, making it challenging for the study psychiatrist to change and adjust therapy. Finally, the absence of a questionnaire such as the Fibromyalgia Impact Questionnaire might have better addressed the impact of this model of care on disease management.

## 4. Conclusions

In conclusion, our feasibility study on the interprofessional model of care has important implications for the management of FM at the community level. Given the promising results on outcomes for patients, a program offered by an interprofessional team has the potential to improve outcomes for FM patients. Furthermore, integrated care is important which was accomplished through the “team huddles” as opposed to care offered in parallel with the different health care providers. The model of care also has the potential to provide family physicians with a structured management pathway and reduce the need for specialist interventions. Further research, however, is needed to determine the effectiveness of this model of care with a larger cohort of patients. In our continued program of research, we are working to continue to ensure family physician involvement as a key HCP to address FM. With continued support from the community and the local health authority, this model of care holds promise in being sustainable and adaptable. The success of this feasibility study is evidence that such comprehensive models of care hold great promise for the long-term management of patients suffering from FM and other chronic illnesses.

## Figures and Tables

**Table 1:** Description of the interventions in the model of care.

Intervention	Frequency	Components
Exercise	One-hour sessions, twice a week	Initial baseline assessments which consisted of a review of medical history, previous activity levels, and a musculoskeletal screen for barriers to exercise
		Developing individual exercise plan that consisted of 4 components: endurance training (treadmill, upright and recumbent bikes, and a recumbent stepper), resistance training (weight machines, free weights, cable machines, and elastic band resistance), flexibility training (floor exercise, BOSU ball, half rolls, and wobble board), and balance training (basic stretches)

Rheumatology follow-up and FM education	One-hour group session, once weekly	Education, follow-up, and support for the patients involving techniques such as pacing, sleep hygiene, and approach to a healthy life style and weight loss

Psychiatric assessment	One time with follow-up as required	Assessment for mood and sleep disorders with pharmacological intervention

Pain education	Two-hour group sessions, twice in 10 weeks	Providing patients with a better understanding of pain mechanisms, perception of pain, and practical solutions in pain self-care

Pain group (Arthritis Society)	Two-hour group session, once in 10 weeks	A peer-led support group from the Arthritis Society provided the patients with a session in pain self-management

Dietitian	One-hour group session, 3 times in 10 weeks	Discussion of general dietary goals and advice

**Table 2:** Patient questionnaires.

Type	Questionnaires	Dimensions
Patient information	Demographics	Age, sex, educational status, employment status, combined household income, ethnicity, current medications, date of FM diagnosis, other morbidities

Pain	Brief Pain Inventory (short form) (BPI)	Intensity, Interference [21]
	Survey of Brief Attitudes of Pain (SOPA)	Solitude, emotions, cure, control, harm, disability and medication [22]
	Pain Catastrophizing Scale (PCS)	Rumination, magnification, and helplessness [23]

Depression and anxiety	Hospital Anxiety and Depression Scale (HADS)	Anxiety and depression [24, 25]

Quality of life	EQ-5D	Mobility, self-care, usual activities, pain/discomfort, anxiety/depression [26, 27]
	Multidimensional Assessment of Fatigue (MAF)	Severity and distress [28]

Function	Sheehan Disability Scale (SDS)	Family impairment, days lost, and days of unproductivity [29]

Sleep	Sleep Scale-Medical Outcome Scale (MOS-SS)	Disturbance, adequacy, somnolence, and sleep problem index [21, 30]

Patient satisfaction	Patient Assessment of Chronic Illness Care (PACIC)	Patient activation, decision support, goal setting, problem solving, and follow-up [31, 32]

**Table 3:** Patient employment and comorbidities.

Patient employment		Patient comorbidities	
Retired	37%	Arthritis	72.7%
Part-time employed	27%	Depression	72.7%
On disability benefits	18%	Chronic pain	63.6%
Unemployed	9%	Headaches	63.6%
Full-time employment	9%	Surgeries	72.7%
		Anxiety	54.5%
		Other conditions	45.4%

**Table 4:** Analysis of patient health questionnaires.

Scales and dimensions	3 months (*n* = 11)		6 months (*n* = 9)		12 months (*n* = 8)	
	Change of median/mean from baseline	Significance (*P* value)	Change of median/mean from baseline	Significance (*P* value)	Change of median/mean from baseline	Significance (*P* value)
	*Brief Pain Inventory*					
Intensity	0.50	0.24	0.00	0.29	−1.5	0.31
Interference	−1.00	0.55	−1.00	0.44	−1.00	0.40

	*Survey of Pain Attitudes*					
Solicitude	3.00	0.07	−3.80	0.80	5.00	0.09
Emotions	0.00	0.72	−9.00	0.55	1.00	0.35
Cure	1.00	0.80	−8.40	0.28	−0.50	0.53
Control	1.00	0.68	−8.00	0.95	5.00	0.18
Harm	0.00	0.63	−7.50	0.68	0.00	0.58
Disability	0.00	0.17	−5.12	0.48	0.50	0.33
Medication	0.00	0.63	−5.67	0.89	−1.50	0.67

	*Medical Outcome Study Sleep Scale*					
Sleep disturbance	−15.91	0.03^*∗*^	−22.61	0.07	−9.48	0.11
Sleep adequacy	12.73	0.26	12.12	0.15	−9.55	0.85
Somnolence	−24.85	0.01^*∗*^	−6.40	0.62	−17.05	0.17
Sleep Problem Index II	−9.75	0.02^*∗*^	−8.10	0.23	11.74	0.03^*∗*^
Hours slept	0.59	0.06	0.38	0.22	0.45	0.31

	*Hospital Anxiety and Depression*					
Anxiety	−0.91	0.17	−1.76	0.20	−0.53	0.53
Depression	−0.64	0.59	−0.58	0.44	0.09	0.48

	*Pain Catastrophizing Scale*					
Rumination	−0.64	0.32	−1.33	0.48	0.33	0.75
Magnification	−0.10	0.81	−0.93	0.48	−0.44	0.59
Helplessness	−0.36	0.92	−1.33	0.44	0.00	0.44

	*Sheehan Disability Scale*					
Family impairment	0.62	0.14	0.16	0.34	−1.15	0.71
Days lost	−0.38	1.00	1.09	0.78	−0.17	0.80
Days of underproductivity	0.81	0.71	0.54	0.46	−0.76	0.83

	*Multiple Assessment of Fatigue*					
Severity	−1.37	0.02^*∗*^	−0.67	0.14	−1.06	0.03^*∗*^

	*Patient Assessment of Chronic Illness Care*					
Total score	1.29	0.01^*∗*^	0.42	0.21	0.10	0.78

	*EQ−5DL*					
Total score	−0.46	0.02^*∗*^	0.03	0.32	−0.01	0.46

^*∗*^ *P* value < 0.05.
